# Phase Transitions in Spatial Connectivity during Influenza Pandemics

**DOI:** 10.3390/e22020133

**Published:** 2020-01-22

**Authors:** Nathan Harding, Richard Spinney, Mikhail Prokopenko

**Affiliations:** 1 Centre for Complex Systems, Faculty of Engineering, The University of Sydney, Sydney, NSW 2006, Australia; richard.spinney@sydney.edu.au (R.S.); mikhail.prokopenko@sydney.edu.au (M.P.); 2 Marie Bashir Institute for Infectious Diseases and Biosecurity, University of Sydney, Westmead, NSW 2145, Australia

**Keywords:** critical dynamics, epidemiology, phase transitions, agent-based modelling, epidemic modelling

## Abstract

We investigated phase transitions in spatial connectivity during influenza pandemics, relating epidemic thresholds to the formation of clusters defined in terms of average infection. We employed a large-scale agent-based model of influenza spread at a national level: the Australian Census-based Epidemic Model (AceMod). In using the AceMod simulation framework, which leverages the 2016 Australian census data and generates a surrogate population of ≈23.4 million agents, we analysed the spread of simulated epidemics across geographical regions defined according to the Australian Statistical Geography Standard. We considered adjacent geographic regions with above average prevalence to be connected, and the resultant spatial connectivity was then analysed at specific time points of the epidemic. Specifically, we focused on the times when the epidemic prevalence peaks, either nationally (first wave) or at a community level (second wave). Using the percolation theory, we quantified the connectivity and identified critical regimes corresponding to abrupt changes in patterns of the spatial distribution of infection. The analysis of criticality is confirmed by computing Fisher Information in a model-independent way. The results suggest that the post-critical phase is characterised by different spatial patterns of infection developed during the first or second waves (distinguishing urban and rural epidemic peaks).

## 1. Introduction

A comprehensive understanding of complex spreading patterns of epidemics and pandemics is of key importance for efficient mitigation and effective response strategies, providing insights into timely and focussed interventions at national and regional levels. Understanding the spatial dispersion of these epidemic spreading patterns may provide insight into early warning and intervention strategies [1]. Specifically, one may be able to find key spatial factors which allow understanding of epidemic spread more deeply [2]. One feature which is of interest in this pursuit is the spatial connectivity of regions with sufficiently high levels of infection, which reflect both geographic and epidemiological features [3]. Such spatial connectivity is not static, but rather changes over time in response to the developing epidemic, highlighting the need for a high-resolution analysis of complex epidemic diffusion patterns. In particular, several recent studies of spatiotemporal dynamics and spatial synchrony of influenza epidemics contrasted the phenomena of both hierarchical and wave-like spreads in different geographical contexts, using the notion of synchrony which is defined as the variance in the timing of the epidemic peaks across different local communities [4,5]. Both studies found that hierarchical spread, characterised by high synchrony across communities of similar sizes, is much more established than wave-like diffusion, which is associated with high synchrony between communities with low pairwise geographical distance. This suggests that the regional spread of a disease depends more heavily on population distribution and flow rather than the geographic distance alone. Spatial synchrony was also implicated in the bimodal pattern of influenza pandemics characterised by two distinct waves: the first wave is observed in highly-urbanised residential centres where the pandemic first reaches a nation (e.g., near international airports), whilst the second wave is observed in sparsely connected rural regions [6].

In this paper, we aimed to investigate how the spatial patterns contribute to the connectivity at different time points corresponding to turning points in the epidemic (e.g., peaks in prevalence, appearance of the second epidemic wave, etc.). This investigation will be carried out within a large-scale agent-based model of influenza pandemics in Australia, enabling the high-resolution simulation of diverse epidemic and pandemic scenarios, and extending beyond the limited capability of canonical meta-population and mean-field approaches. Furthermore, we shall relate the spatial connectivity of simulated pandemics to epidemic thresholds and critical phenomena, using both percolation theory and model-independent information-theoretic measures.

One major challenge in epidemic modelling is a faithful recreation of the demographic features of the affected population in a way that makes the modelling results transferable to a real-world context. Agent-based models (ABM) have been demonstrated to be particularly effective in tracing epidemics under different “what-if” scenarios and comparing possible interventions [6,7,8,9,10,11,12,13]. The primary reason for this success is that these agent-based approaches allow for high-resolution population and mobility data to be effectively incorporated into simulation and analysis. As high-resolution data continue to become more easily accessible, this strength needs to be complemented by rigorous theoretic frameworks and analytic techniques. Traditional compartment-based approaches (e.g., SIR models) struggle to incorporate these high-resolution datasets as easily as ABM models, relying on assumptions of well-mixed homogeneous populations describable by just a few parameters defining their interactions [14,15,16]. Instead, ABM offers scalable simulation frameworks, while providing further spatially refined data for a detailed analysis informed by percolation and information theories.

The studies of critical phenomena have already significantly contributed to computational epidemiology. Typically, this analysis focuses on a critical epidemic threshold, above which epidemics develop and persist and below which epidemics die out. This dates back to the seminal study of Kermack and McKendrick [15] who identified the ‘threshold behaviour’ of the model. This type of analysis has been extended to many complex models and the current discourse is generally based on the basic reproductive ratio $R_{0}$ [14,17,18,19,20,21,22,23,24,25,26], i.e., the number of secondary cases arising from a typical primary case early in the epidemic, as well as the attack rate [7,27,28], i.e., the proportion of a population infected by the conclusion of an epidemic. For example, recent studies considered critical behaviour in the spatial spread of epidemics in statistical-mechanical terms, interpreting key epidemic variables, such as the reproductive ratio, thermodynamically [29], while also identifying phase transitions related to dynamic mobility and interaction patterns [30]. We extend this approach, by connecting an ABM of influenza pandemics in Australia, which has been validated previously, with a broader theoretical framework.

What makes this connection more interesting is the fact that Australia has a number of demographic, geographic, and ecological features which non-trivially affect socio-economic connectivity and mobility patterns [3,31,32,33,34,35]. In contrast to many other countries with a more even spatial population distribution, Australia comprises a relatively small number of densely populated urban centres distributed along the coastline, sparsely connected to many more low-density inland towns and rural/regional communities. This particular population distribution has been implicated in Australia’s highly bimodal epidemic curves, with modes associated with its urban, and rural communities [6].

In this study, we present a general method identifying critical regimes in spatial connectivity during epidemics. This analysis is performed using data generated by AceMod, the Australian Census-Based Epidemic Model [5], an agent-based simulation of influenza epidemics in Australia. This could, in principle, be performed with analogous real or simulated spatial data; however, as AceMod incorporates high-resolution population and mobility data provided by the Australian Bureau of Statistics (ABS), this allows our connectivity analysis to be applied in a real-world context. We examined the spatial extent of epidemic spreading at two key points of the simulated epidemics, while varying infectivity parameters. While the disease progression across the regions is a result of the ABM simulation, quantifying the spatial connectivity of infection using the methods of percolation theory is an important additional step, making it one of the main contributions of this study. This was achieved with the use of percolation theory to measure connectivity, alongside the use of Fisher Information as a model-independent measure pinpointing phase transitions. Specifically, we quantified the mean cluster size to which a randomly selected “infected” location belongs [30,36], denoted the ‘infection connectivity’ in order to identify prominent clustering patterns during these multiple epidemic peaks of an Australian epidemic. Finally, we investigated the relationship between the infection connectivity at each of the epidemic peaks and the infectiousness of the disease.

## 2. The Simulation Framework

AceMod [5,6] is an agent-based modelling simulator for studying the spatial spread of influenza in Australia. Despite its focus on modelling the Australian population, it is methodologically similar to other well established agent-based models [7,8,12,28]. At a high level, AceMod can be described as follows: The model is initialised by stochastically generating a surrogate population of Australia, in this study, based on the 2016 Census. We consider a plausible scenario of a pandemic strain of influenza which initially arrives in Australia via air traffic from overseas due to the presence of a global outbreak which constitutes the seeding conditions of the simulation. The arrival of these infected individuals maintains a stream of new infections at each time step in proportion to the air traffic of each local airport. These infections occur probabilistically in individuals within a 50 km radius of the airport. Upon reaching Australia, the disease continues to spread through households, communities, workplaces and schools based on pertinent local infection parameters and the natural history of the disease. In spreading through Australian society, the simulator separates each day into two ‘phases’ a ‘daytime’ and ‘night-time’ phase. These two phases consider distinct mixing behaviours with the night-time phase incorporating household and other local interactions within residential communities and the daytime phase incorporating interactions which arise within workplaces and schools [5,6] (See Section 2.2 or [5] for more detail).

AceMod uses 3 key levels of resolution which are important for both the simulator and the spatial analysis presented within this paper. As an agent-based model, the lowest level of resolution of AceMod are individual agents, representing individual members of Australian society. The census data upon which AceMod is built is defined on local regions called ‘Statistical Area 1’ (SA1) and ‘Statistical Area 2’ (SA2), which are defined by the Australian Statistical Geography Standard [37]. The SA1 is the lowest level of resolution in the simulator above the level of individual agents and is designed by the ABS to ‘maximise the spatial detail available for the census’ [37]. Above this level is the SA2—a key level of resolution of the Australian census which encompasses multiple SA1s, and is instead designed to “reflect functional areas that represent a community that interacts together socially and economically”. Of the two, the SA2s will be the primary object of our spatial analysis when focusing on the connectivity of community areas.

The AceMod simulator has two distinct aspects which are extremely important in faithfully recreating the dynamics of a realistic Australian pandemic scenario. These distinct aspects are common to all agent-based frameworks for epidemic modelling and can be broadly separated into ‘population description’ and ‘disease description’ which we detail in the following sections.

### 2.1. Population Generation

Population generation occurs at the SA1 level. For each SA1, local statistics regarding population (age, sex, employment status) and housing (household size and composition) are used to construct probability distributions representing the population of each of these areas. These distributions are then used to generate households which, when aggregated, represent the real distribution of households within the region. Once households have been generated, agents are generated stochastically to fill each specific household (e.g., a single parent family with three members will generate one adult agent and two child agents). These household and community layers form the contact network for the ‘night-time transmission’. Once all agents have been generated, the Travel-to-Work (TTW) data are used to assign workplaces to the generated adult population. The TTW data contains a list of home and work region pairs along with a number of individuals associated with the pair. Therefore, in order to occupy each work location, the population generation procedure moves sequentially through the TTW data, selecting the appropriate number of working age individuals without working locations from the home location, assigning them to work in the given work region. While the locations and number of students of schools are precisely known, due to a lack of high-resolution data about school attendance, the school-aged population cannot be assigned to schools in the same way. Instead, they are assigned to schools stochastically, primarily based on the radial distance with individuals significantly more likely to attend school at geographically closer locations (see [5] for full details of this student allocation process).

### 2.2. Disease Description

**Figure 1:**
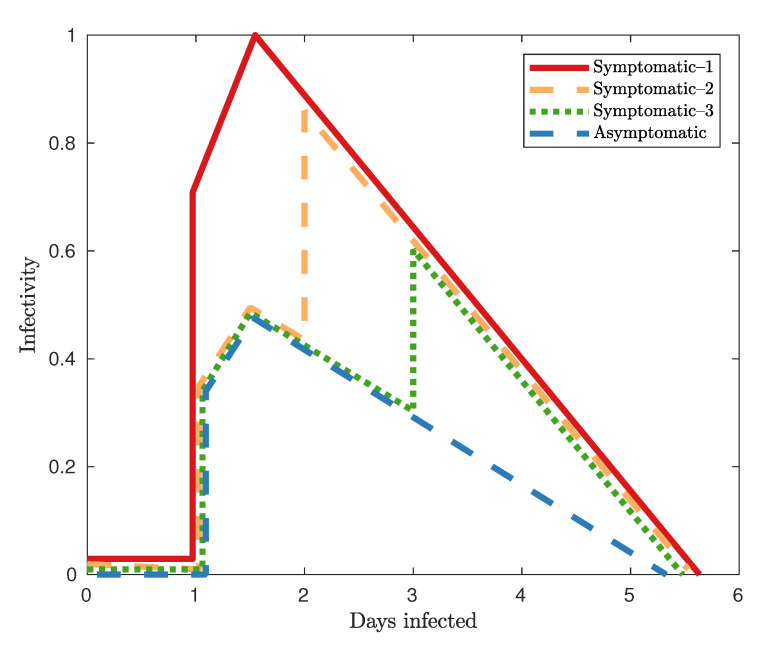
The natural history of the disease used in the AceMod simulator [5]. The infectivity of an agent is modelled with an initial linear increase, followed by a linear decrease as the host recovers. Time 0 indicates the time at which an individual is infected. For the first day of infection, all infected individuals are in the Latent state and are not infectious. 33% of cases are Asymptomatic, following the Asymptomatic infectivity curve (in blue). Of the Symptomatic individuals, 30% of individuals become fully infectious on day 1 (solid line), 50% on day 2 (orange dashed line) and 20% on day 3 (blue dotted line).

Secondly, we describe the progression of the disease itself. This can be separated into two key components: the natural history of the disease and the transmission model of the disease. The natural history of the disease describes the progression of the disease within an individual after infection, whereas the transmission model comprises the rates at which the epidemic spreads between hosts. We define a number of states which characterise the status of an agent with respect to the epidemic: Susceptible, Latent, Asymptomatic Infectious, Symptomatic Infectious and Recovered. The progression of disease within a host occurs as follows: when an individual becomes infected, they transition from the Susceptible state to the Latent state, where they are infected, but are not yet able to infect others. After the Latent state, individuals transition to either the Symptomatic or Asymptomatic Infectious state. The primary difference between these infectious states is the level of infectiousness with Symptomatic individuals being twice as infectious as Asymptomatic individuals [12] at the same time of disease progression (see Figure 1). After an individual’s infectious period, they recover with immunity, transitioning to the Recovered state. For extensive details of the natural history of the disease, see [5].

The primary mechanism we are interested in in epidemic models is transmission of the disease from infected to uninfected hosts. As such, we consider a random variable $X_{i}\left(t\right)$, the infection status *X* of an individual *i* at time *t*. The primary object of interest to an agent-based model of epidemics is the probability that an individual who is susceptible at time t−1 becomes infected at time *t*$p_{i}\left(t\right)=P\left(X_{i}\left(t\right)=L\mathrm{ATENT}|X_{i}\left(t-1\right)=S\mathrm{USCEPTIBLE}\right)$. In the AceMod simulator, this probability is formed by multiplying transmission probabilities across all mixing groups $G_{i}\left(t\right)$ which individual *i* interacts with in time period *t*. Each group $g\in G_{i}\left(t\right)$ is associated with a set of static agents $A_{g}$. The infection probability is thus computed as

(1) $$p_{i}\left(t\right)=1-\prod_{g\in G_{i}\left(t\right)}\left[\prod_{j\in A_{g}\backslash i}1-\rho_{j\to i}^{g}\left(t\right)\right],$$

where $\rho_{j\to i}^{g}\left(t\right)$ is the probability that individual *j* infects individual *i* at time *t* in the mixing group *g*. It is particularly important that the parameters describing the infection spread are realistic; therefore, we utilised transmission probabilities from empirical studies when available to characterise these transitions [5,7,38]. Unfortunately this is not always possible and when these direct transmission probabilities are not available, relative transmission probabilities are derived from contact probabilities [8,38]. These are constructed as the product of a conditional probability of a contact probability $q_{i\to j}^{g}\left(t\right)$ and a scaling parameter, calibrated to known attack rates. We then consider a free parameter κ which multiplies all infection probabilities equally. In this way, we are able to change the infectiousness of the disease while preserving relative infection rates and as a result, vary a key epidemic parameter, $R_{0}$—the basic reproductive ratio, a measure of the number of secondary cases caused by a typical primary case which occurs early on in the epidemic [17].

### 2.3. Epidemic Synchrony and Bimodality

AceMod has been utilised in a number of studies simulating Australian epidemics. The results of these studies highlighted the role of the synchrony of local epidemic curves, as well as the bimodality present in the global epidemic curves.

Broadly speaking, synchrony in an epidemic system refers to the phase coherence of incidence curves in specified locations [4]; in general, it is defined as the inverse of the variance of a collection of peak times. In a previous analysis of simulated epidemics produced by AceMod [5], synchrony was used to differentiate between a wave-like and hierarchical spread. In this analysis, synchrony was characterised in two ways: spatial synchrony and hierarchical synchrony. Hierarchical synchrony was defined as the inverse of the variance of the peak times within similarly sized regions, whereas spatial synchrony was defined in terms of mean pairwise synchrony between regions of similar radial distance from one another. Cliff et al. [5] identified a strong hierarchical synchrony between regions of similar sizes, providing evidence that hierarchical spread of the epidemic is more prominent than wave-like diffusion. The study also noted that the hierarchical synchrony tended to increase as the infectiousness of the disease was increased.

Of principal importance to our study are the results of Zachreson et al. [6] which identified a distinct bimodality in the global Australian pandemic curve. This bimodality was quantified by grouping SA2s in terms of their local peak prevalence dates, allowing each SA2 to uniquely be attributed to a mode of the epidemic, identifiable with an epidemic wave. The primary mode of the epidemic is comprised almost exclusively of SA2s in the urban centres of Australia, whereas the secondary mode primarily consists of rural SA2s. Zachreson et al. identified that this bimodality was becoming more pronounced based on changes in population density and demographics across multiple years (2006, 2011 and 2016), attributed to urbanisation and increasing international air travel [6].

**Figure 2:**
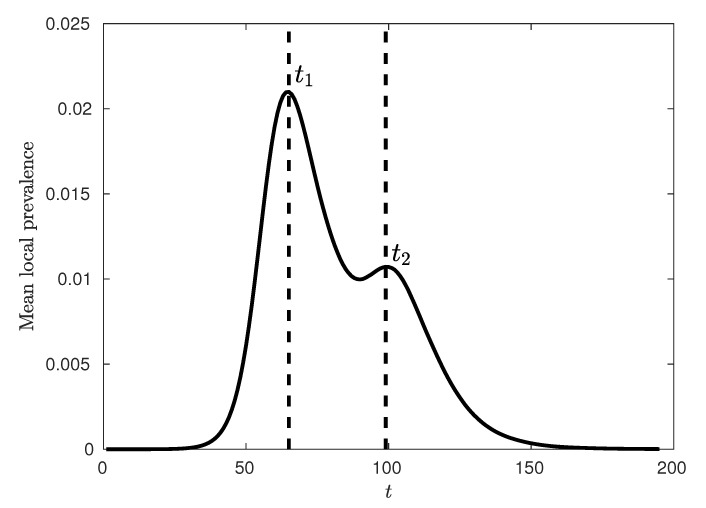
A typical epidemic curve tracing the average local prevalence fraction for κ=2.5. Each of the dotted lines indicate the times of the two local maxima, $t_{1}$ and $t_{2}$ (urban and rural peaks of the epidemic).

**Figure 3:**
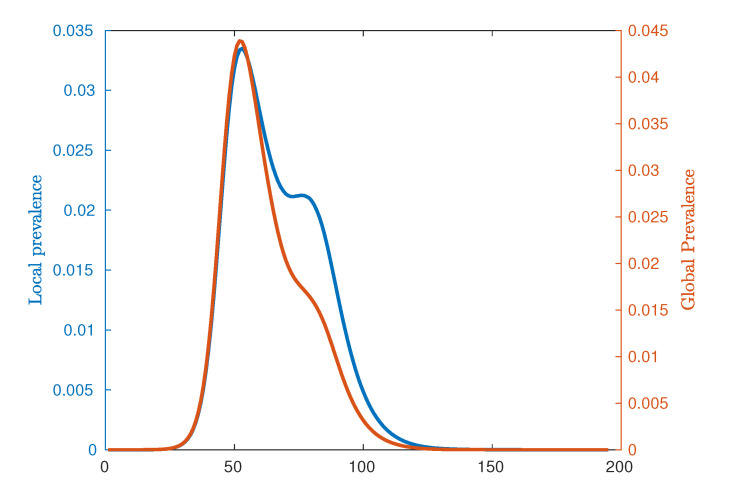
Comparison of global and averaged local prevalences for κ=3.33. Local measures accentuate the presence of the second (rural) peak due to relative differences in population between urban and rural locations.

The analysis presented in this paper is focussed on each of these epidemic peaks, aiming to identify salient spatial features of the entire system at each of these key time points. Figure 2 demonstrates a typical epidemic curve in terms of the mean local prevalence fraction, with the location of the first (urban) and second (rural) peaks of the epidemic marked as $t_{1}$ and $t_{2}$ respectively. Figure 3 contrasts the global epidemic curve and the mean local prevalence fraction. Mathematically, the global prevalence is defined as $\sum_{i}I_{i}/\sum_{i}N_{i}$ whereas the mean local prevalence is defined as $\sum_{i}I_{i}/N_{i}$, where $I_{i}$ is the number of infected individuals at location *i* and $N_{i}$ is the number of individuals in location *i*. Following [6], we observe that the mean local prevalence identifies the urban and rural peaks more clearly than the global prevalence, as it better accounts for contributions from smaller (rural) areas, while the global curve obfuscates the location of the secondary peak.

## 3. Methods

### 3.1. Percolation Phase Transitions

**Figure 4:**
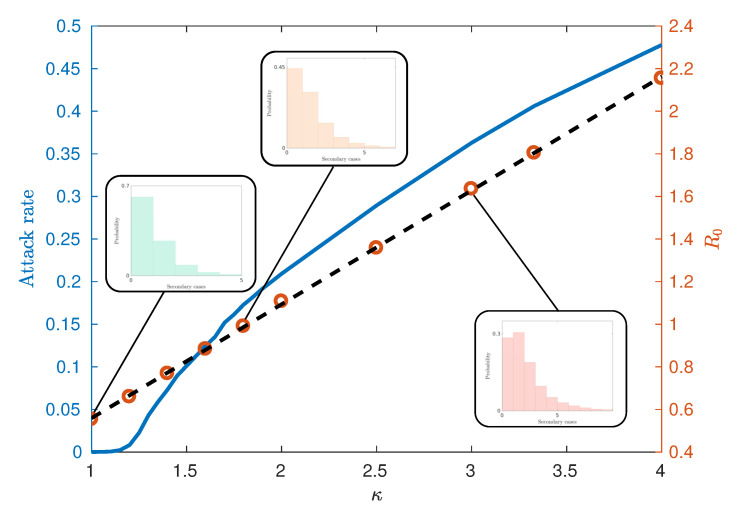
The relationship between the attack rate and κ (blue curve) and the relationship between the critical threshold $R_{0}$ and the scaling parameter κ (circles) estimated from 65,000 initial seeds. The standard error of the mean is smaller than the marker size. The black dotted line indicates the line of best fit $R_{0}=0.5345\kappa +0.0247$ with an $R^{2}$ value of 0.9998. This gives a critical value of $R_{0}^{*}\approx 0.67$ for $\kappa^{*}$ between 1.15 and 1.25. Shown inset are the distributions of secondary cases from random primary cases which are used in calculating $R_{0}$.

Broadly, a percolation phase transition is the rapid change of a system from a phase characterised by disconnected, disparate, clusters into one characterised by small numbers of large highly connected clusters upon a small variation in a *control parameter*, which parameterises some system dynamics, at its *critical value*. We seek to apply this notion to epidemics by identifying clusters of highly infected geographical regions, where clusters are defined as contiguous regions of elevated infection. We consider regions with elevated infection levels to be those with local infection prevalence fraction above the mean over all such geographic locations at the time of evaluation (the peaks of the urban and rural waves of the epidemic). Our control parameter is then taken to be the infectiousness parameter κ. The reasons to choose a relative threshold are twofold firstly, it allows the interpretation of high infection to be considered independently from the issue of population density. Secondly, it allows the framework presented to be applied across a range of infectiousness parameters, where the total number of infections across the simulation (attack rate) has a strong dependence on the infectiousness (κ) (See Figure 4).

In order to identify a percolation phase transition, we aimed to capture the emergence of this large connected component utilising a simple measure $\hat{r}=\frac{r}{N}$, where *r* is the size of the cluster to which a randomly selected site belongs [36] and *N* is the total number of sites such that $\hat{r}$ does not depend on system size. We may then consider $p(\hat{r})$, the probability that a randomly selected site belongs to a cluster of normalised size $\hat{r}$. The mean of this distribution $\langle \hat{r}\rangle$ has been used as an order parameter to identify percolation phase transitions in epidemic dynamics [30]. A percolation transition would then be characterised by a discontinuous change in $\hat{r}$ as κ is varied.

In our analysis, we used the simulated epidemic generated by AceMod as the background process which, at a specific time point, produces a snapshot of an epidemic spread using the geographic adjacency of SA2s as the underlying topology. Importantly, the geographic SA2 adjacency which we used in our percolation analysis of the infection spread should not be confused with the adjacency of the underlying direct interaction network, defined at the individual agent level in terms of both travel to work and school travel. This distinction is significant because although the true transmission pathways may generally not be known, the geographic adjacency of SA2s can always be established, allowing for application to systems involving real data.

We traced the spatial connectivity of the infection based on the spatial pattern of infection prevalence at the two key time points typical for the Australian pandemic scenarios. These points (illustrated in Figure 2) coincide with the urban and rural peaks of the epidemic respectively [6] (see Section 2.3).

### 3.2. Fisher Information

Phase transitions are associated with large-scale transformations of a system and thus, are reflected in the probability distributions of the model. Consequently, they may be identified in a model-free manner in terms of this probability distribution. The primary way to do so is with a quantity called the Fisher information, $F_{X}\left(\theta \right)$, which may be understood as the information that a random variable *X* (in our case the state of the epidemic I) contains about a parameter θ (in our case the control parameter κ). The Fisher Information is defined as the expectation

(2) $$F_{X}\left(\theta \right)=E\left[{\left(\frac{\partial }{\partial \theta }\log P\left(X;\theta \right)\right)}^{2}\right]$$

where P(X;θ) is the probability distribution of X conditioned on θ. Informally, the Fisher information quantifies sensitivity of the distribution modelling the random variable *X* to changes in the parameter θ. Consequently, the large-scale changes in the system state associated with a phase transition are accompanied by a large-scale change in the distribution which causes the Fisher Information to peak. When this change is discontinuous, the Fisher information then diverges. Equivalently, since the Fisher information quantifies the information *X* provides about θ, we can interpret this as a vanishing of uncertainty in the value of θ given observations of *X* around the critical point. The association between phase transitions and the Fisher information is well established [29,39,40,41,42,43,44,45,46,47], with the divergence at criticality due to a direct relationship between Fisher information and the rate of change of a corresponding order parameter [44].

In many cases, the full joint probability distribution P(X;θ) is impractical to work with in this context, due to high dimensionality. However, as our analysis relies on peaks/divergences in the Fisher information rather than its exact value, we can exploit the inequality $F_{T}\left(\theta \right)\leq F_{X}\left(\theta \right)$, where *T* is a (more tractable) statistic of *X* with equality holding if and only if *T* is a sufficient statistic of *X*. Then, under some relatively weak assumptions, a divergence in $F_{T}\left(\theta \right)$ implies a divergence in $F_{X}\left(\theta \right)$ and thus, the existence of a phase transition. In our context, *T* is then taken to be the percolation statistic $\hat{r}$.

## 4. Results

In this section, we present an analysis of the spatial characteristics of the simulated Australian epidemics. We begin, however, with a basic analysis of common epidemic characteristics, specifically, the dependence of attack rate, the basic reproductive ratio $R_{0}$ and the timing of the urban and rural peaks on the infectiousness parameter κ, used in AceMod.

As infectiousness (defined here through κ) increases across a population, the number of secondary cases caused by an average primary case, characterised by $R_{0}$, also increases. Classically, the behaviour of epidemics is then characterised by two distinct regimes separated by a critical value of $R_{0}=R_{0}^{*}$. Above this threshold (with higher infectiousness) small initial infections grow into epidemics whilst below this threshold (with lower infectiousness), initial infection levels decay away and epidemics do not occur. Such a critical value of $R_{0}$ then implies that there is a critical value of $\kappa =\kappa^{*}$ used in the AceMod simulations. Consequently, to characterise the behaviour generated by AceModns, we first seek to quantify the relationship between κ and $R_{0}$, then, in conjunction with the attack rates, identify their critical values.

There are several methods for computing $R_{0}$ from simulated data in the literature attempting to capture the predominant spreading behaviour in subtly different ways [7]. Figure 4 illustrates an observed linear relationship between κ and $R_{0}$ calculated using the ‘random index case’ method [7]. This method involves randomly selecting individuals from the population in an unbiased way, infecting them and observing the number of secondary infections produced from the single primary case. This is in contrast to other methods for calculating $R_{0}$ such as the ‘attack rate pattern weighted index case method’ [7], which biases the infection of individuals based on relative attack rates between communities.

Figure 4 also demonstrates the relationship between the attack rate (the proportion of the population who have been infected by the end of the epidemic) and κ, from which we may estimate the critical values of κ and $R_{0}$. We observe that for this model, the critical value $\kappa^{*}$ lies in the interval (1.15,1.25), corresponding to $R_{0}^{*}\approx 0.67$. Notably, this critical value differs from models which assume continuously mixing populations such as compartmental models and metapopulation models [15,16], where $R_{0}^{*}=1$. However, such deviations are commonly observed within other agent-based or network-based approaches with heterogeneities and imperfect mixing [7,8,18]. The reasons for observing epidemics where $R_{0}^{*}<R_{0}<1$ can be attributed to several factors. The predominant reason is the existence of super-spreaders within the highly urbanised communities and certain agent categories (see the fat-tailed distributions of Figure 4 inset), which may be initially infected and contribute to further spread beyond a vanishing epidemic. Finally, the choice of the ‘random index case’ method used to calculate $R_{0}$ tends to produce lower values than other methods such as the ‘attack rate pattern weighted index case’ method [7].

**Figure 5:**
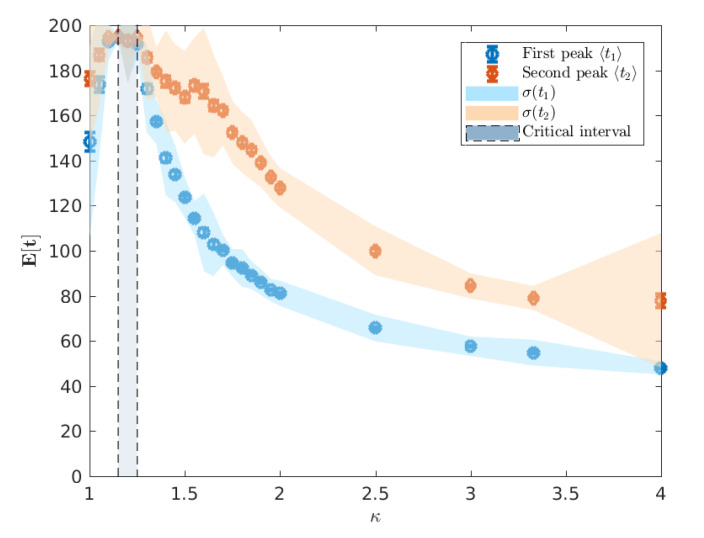
Mean peak times $E[t_{1}]$ and $E[t_{2}]$ respectively calculated from 100 runs. The AceMod simulator caps simulations at 195 days and so low values of κ indicate that the epidemic has not developed sufficiently within the 195 day period. The vertical shaded region $\kappa^{*}\in \left[1.15,1.25\right]$ marks the critical interval of κ for which we observe non-vanishing attack rates (cf. Figure 4). The shaded region around the points indicate the standard deviation in the distribution of peak times $t_{i}$ while the error bars indicate the standard error of the mean $E[t_{i}]$.

Observation of AceMod simulation data indicates that the infectiousness of individuals controls not only the extent of the epidemic, as measured through the attack rates, but the whole evolution of its progression. Of particular interest is the *timing* of the epidemic which we characterise using the mean of the urban and rural prevalence peaks, $E[t_{1}]$ and $E[t_{2}]$ (see Figure 3). The variation of these mean peak times varying against κ are shown in Figure 5. Importantly, we point out that the AceMod simulator terminates after 195 days. Consequently, regions of the parameter space of κ for which t≈195 indicate the lack of a significant epidemic or the inability to distinguish a second peak due to low global infection.

**Figure 6:**
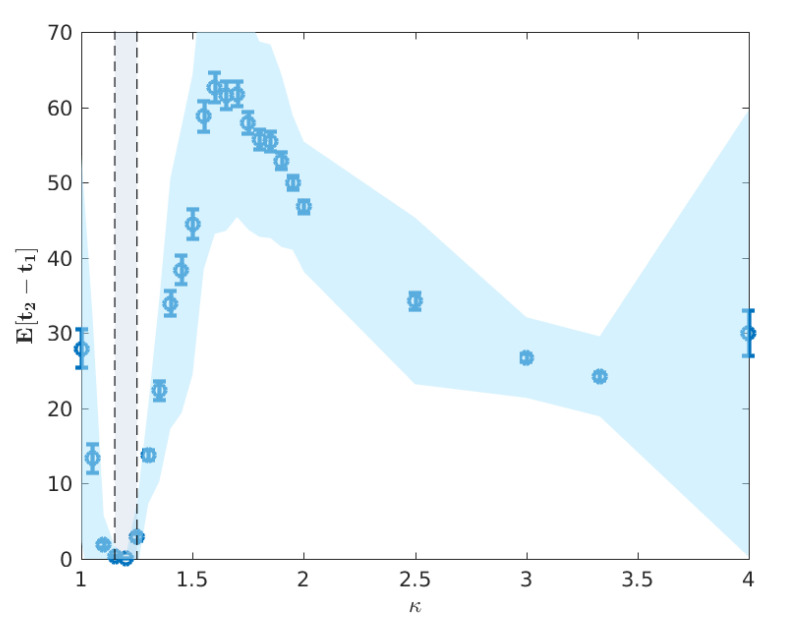
Mean difference in peak times $E[t_{2}-t_{1}]$. Error bars indicate the standard deviation in the difference $t_{2}-t_{1}$, whereas the blue shaded region represents the standard deviation of the samples. The vertical shaded region $\kappa^{*}\in \left[1.15,1.25\right]$ marks the critical interval of κ for which we observe non-vanishing attack rates (cf. Figure 4). Directly after the critical region, we observe a decoupling in the timing of the epidemic peaks, illustrated by the rapid increase in this difference in the post-critical phase.

We note that for κ≲1.10, there is no significant epidemic such that the indicated peaks are dominated by noise. For 1.10≲κ≲1.25, around the critical point, the characteristic lack of clear exponential growth or decay combined with the constant seeding results in nominal peaks in both waves to occur at the time horizon for any reasonable simulation length (here limited to 195 days). More pertinently, as we increase κ beyond the critical interval, the average timing of two peaks begins to decouple, as shown in Figure 6, and we begin to observe two clearly distinguished profiles. In this post-critical phase, κ≳1.25, each profile monotonically decreases, indicating that the respective peak occurs earlier in time for higher values of κ. In order to understand the spatial extent of Australian epidemics, we offer a characterisation in terms of the extent of spatial spread at the two key points of the epidemic: namely, the urban and rural peaks of the epidemic $t_{1}$ and $t_{2}$ [6] (see Section 2.3).

**Figure 7:**
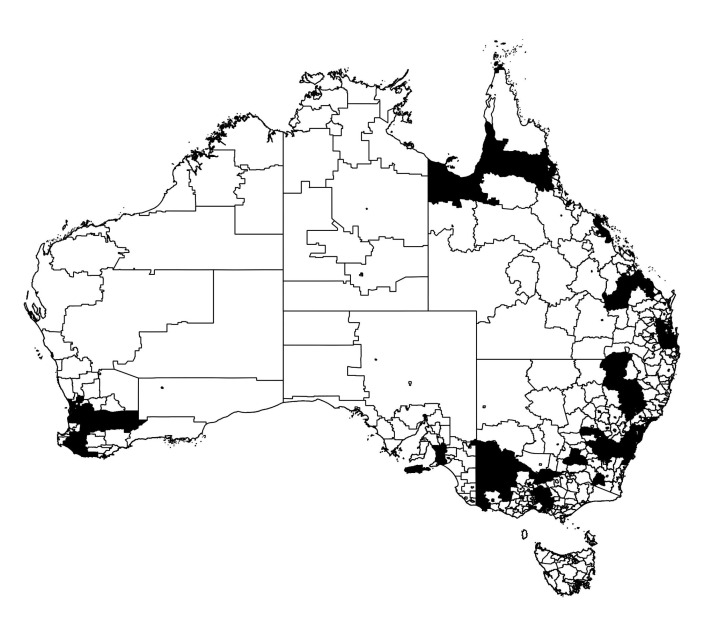
Thresholded infection map of the Australian epidemic at the primary peak of infection of Figure 2. The regions in black represent SA2s with greater than average prevalence fraction $\langle I\left(t_{1}\right)\rangle$, at time $t_{1}$, whereas the regions in white show the areas with infection below this threshold.

**Figure 8:**
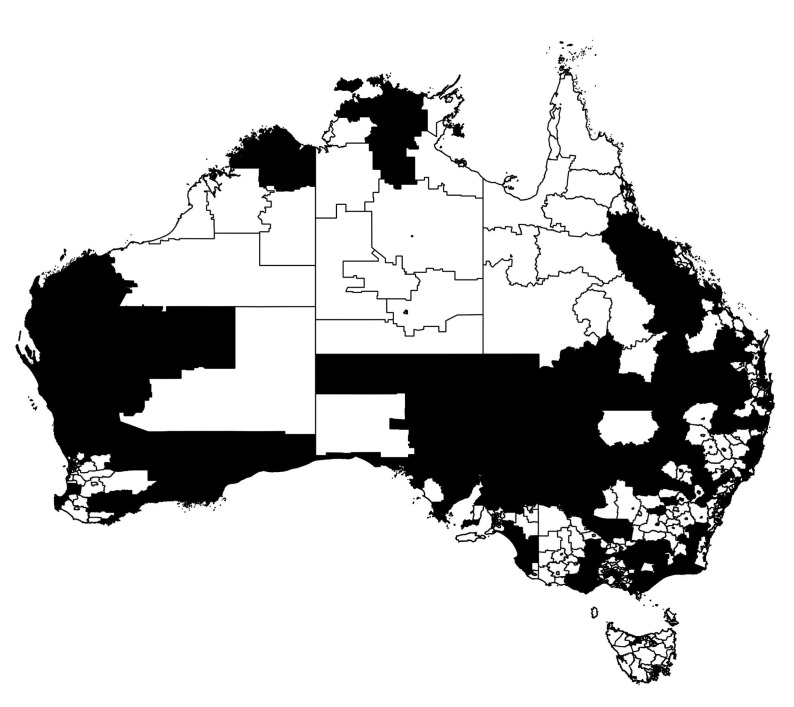
Thresholded infection map of the Australian epidemic at the secondary peak of infection Figure 2. The regions in black represent SA2s with greater than average prevalence fraction at time $t_{2}$, $\langle I\left(t_{2}\right)\rangle$, whereas the regions in white show the areas with infection below this threshold.

In particular, we are concerned with the spread of the epidemic across the extended spatial system, differentiating between highly connected and isolated infected communities, as commonly quantified using percolation measures. In order to utilise such measures, we thus classify each region as either strongly or weakly infected, using the mean prevalence fraction as the threshold for this distinction. The distribution of strongly and weakly infected regions at example times $t_{1}$ and $t_{2}$ are illustrated in Figure 7 and Figure 8, with black and white corresponding to strongly and weakly infected regions, respectively.

The extent to which the epidemic is well connected or isolated, across all population areas can then be characterised by the degree of clustering in the strongly infected regions. This is achieved by (i) identifying spatially contiguous regions of strongly infected locations and identifying them as clusters, (ii) recording all cluster sizes as a (normalized) number of connected regions and (iii) constructing the probability distribution $p(\hat{r})$ that a randomly chosen region belongs to a cluster of normalised size $\hat{r}$. Configurations with larger clusters and thus, larger values of $\langle \hat{r}\rangle$, are more connected than configurations with smaller values of $\langle \hat{r}\rangle$. This quantitative examination can better characterise the actual spatial “infection” connectivity than a simple visual inspection: for example, Figure 7 depicts the state which happens to be more connected than the one shown in Figure 8.

**Figure 9:**
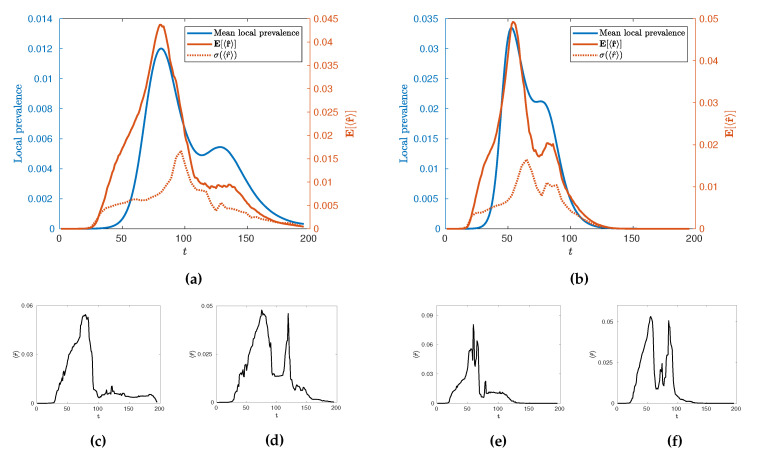
Temporal comparison of local measures of infection the mean local prevalence and $E[\hat{r}]$ for κ=2 (**a**) and κ=3.33 (**b**). We note that the standard deviation of $\langle \hat{r}\rangle$ (dotted line) lags behind E[$\langle \hat{r}\rangle$] and the epidemic curve. We observe a large amount of variability in the connectivity of the secondary wave of the epidemic, whereas the first wave is relatively stable. Underneath each averaged time-series are two extremal examples of the time series $\langle \hat{r}\rangle$(t) where the second wave is very poorly connected (**c**,**e**) or extremely highly connected (**d**,**f**).

In the first instance, we may use this new measure, $\langle \hat{r}\rangle$, to characterise the temporal evolution of the epidemic by calculating its mean value across many realisations, $E[\langle \hat{r}\rangle ]$ as a function of time for different values of κ. Such evolution is illustrated in Figure 9 for κ=2 and κ=3.33 alongside its standard deviation, $\sigma (\langle \hat{r}\rangle )$, and some illustrative individual trajectories for specific extremal realisations of epidemics. Broadly, we see an evolution with two local maxima as with the local prevalence indicating that the increases in local prevalence associated with the urban and rural waves are accompanied by an increase in spatial clustering. The height of the second peak, however, depends on the value of κ with a stronger second peak occurring at higher values. Some insight into this behaviour can be gained by observing the variance of the values and individual realisations of the epidemic. In particular, we see a marked increase in variance in $\langle \hat{r}\rangle$ *after* the initial urban peak, implying that this time is a crucial point in the evolution with regards to the spatial connectivity of the epidemic whereby a great number of qualitatively distinct spatial spreading behaviours become possible. This can then be observed in the example extremal realisations which all have similar rises in $\langle \hat{r}\rangle$ leading to the urban peak, but a range of possible subsequent behaviour which may or may not include a second, rural, peak in clustering. We conjecture that this is due to an inherent sensitivity in the evolution of the epidemic in terms of timing and configuration, when the spread reaches the edge of urban populations. This implies that there are qualitatively distinct ways to spread through rural populations as opposed to the generally uniform spread through urban centres. Interestingly, as κ is increased to 3.33 we see that whilst this variance peak between waves remains similar in size, the variance then subsequently increases as $E[\langle \hat{r}\rangle ]$ increases. This suggests that the expected connectivity of the rural wave is subject to greater variations due to an increase in the number of realisations with both highly connected and highly disconnected rural waves.

The fact that increases in prevalence are associated with increases in spatial clustering immediately presents a question related to the existence of the usual phase transition at $\kappa =\kappa^{*}$ and whether it can be characterised as a *percolation* transition in terms of $\langle \hat{r}\rangle$ alongside its variation with κ more broadly. Explicitly, does the existence of a phase transition between growth and decay from infection seeding imply a percolation transition? Further, given the variation in the behaviour at the rural peak, how does the connectivity and distribution vary with κ at this point in the evolution of the epidemic? To answer these questions, we investigate the behaviour of $\langle \hat{r}\rangle$, both through its mean, $E[\langle \hat{r}\rangle ]$, and the sensitivity of its distribution through the Fisher information, as it varies with κ as evaluated at the urban and rural peaks of the epidemic since these capture (i) the peak in spatial connectivity and (ii) the behaviour during the rural wave.

**Figure 10:**
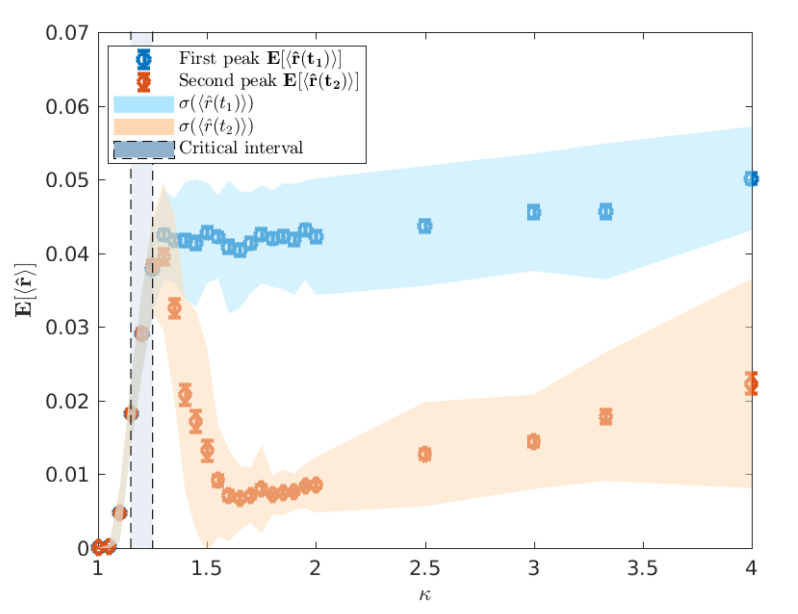
The average E[$\langle \hat{r}\rangle$] over 100 runs, traced across a range of 1≤κ≤4, where $\langle \hat{r}\rangle$ is the normalised mean cluster size to which a randomly selected location belongs at each of the two epidemic peaks. The first peak corresponds to the urban wave of the epidemic whereas the second peak reflects the rural epidemic wave. The error bars indicate the standard error of the mean $\langle \hat{r}\rangle$. The vertical shaded area for $\kappa^{*}\in \left[1.15,1.25\right]$ indicates the critical interval of κ.

First, we plot $E[\langle \hat{r}\left(t_{1}\right)\rangle ]$ and $E[\langle \hat{r}\left(t_{2}\right)\rangle ]$ as functions of κ in Figure 10. As κ increases from 1 towards the critical interval, we observe a rapid increase in $\langle \hat{r}\rangle$ until κ≈1.3 where $E[t_{1}]$ and $E[t_{2}]$ are indistinguishable, indicative of a percolation phase transition. However, as the peaks become increasingly distinguishable in the post-critical regime, there is a distinct difference in the relationship between $\langle \hat{r}\rangle$ and κ when measured at the urban and rural peaks.

The minimum in this profile, around κ≈1.65 (Figure 10), occurs when the difference in mean peak times $E[t_{1}]$ and $E[t_{2}]$ is the largest (Figure 6). We argue that this minimal connectivity, which coincides with the maximum difference in peak times marks the minimum value of κ for which the urban and rural waves of the epidemic are meaningfully separated such that all measured connectivity at $E[t_{2}]$ is a result of the rural wave. In contrast, below this value, the urban and rural waves are, to different extents, overlapping and thus, much of the measured connectivity at $E[t_{2}]$ is attributable to the urban wave. In light of this, it is interesting to note the distinct behaviour measured at $E[t_{1}]$ and $E[t_{2}]$ beyond κ≈1.65. Specifically, we see a reasonably constant connectivity and variance measured at $E[t_{1}]$, but a distinctly increasing connectivity and variance measured at $E[t_{2}]$. This supports the view implied by Figure (Figure 9) where the onset of the urban wave is a consistent, reliable phenomenon, whereas the rural wave is a much more variable phenomenon with many more possible realisations. As κ continues to increase, the relatively low probability of a large connected rural peak gets larger, but with increased variability. This is again observed in Figure 9 where the extremal cases include realisations with no rural peak in spatial connectivity for both measured values of κ.

At this stage, we support the assertion of a percolation phase transition between low and high connectivity configurations using model-free measures of criticality, namely the Fisher Information. We compute the Fisher information using the probability distribution $p(\hat{r})$, implicitly taking $\hat{r}$ as a statistic of the full phase space, instead of the configurational probability distribution defined in terms of the system’s Hamiltonian [44]. In a canonical statistical physics setting, the Fisher information is proportional to the rate of change of the order parameter, being analogous to susceptibility which has a singularity diverging at the critical point. As discussed in Section 3.2, a divergence in this Fisher information based on the statistic $\langle \hat{r}\rangle$ implies a phase transition across the wider state space.

**Figure 11:**
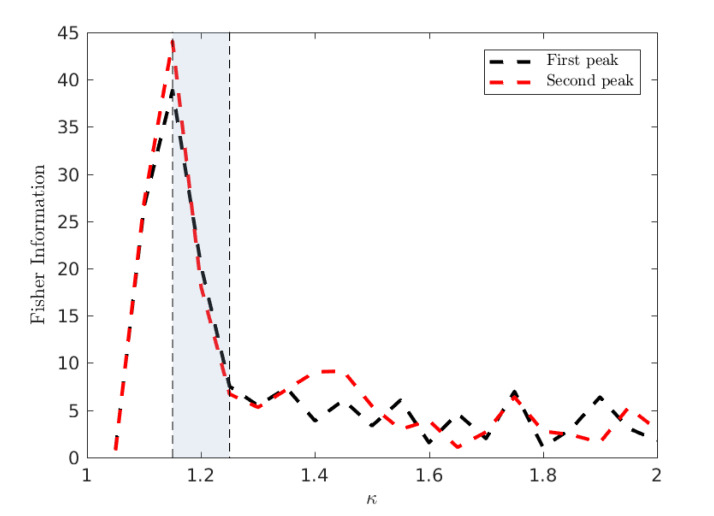
The Fisher information of $\hat{r}$, the cluster size to which a randomly selected site belongs, based on a bin width of 5. The Fisher information identifies the peak observed in E[$\langle \hat{r}\rangle$] concurring with critical interval of κ.

In order to accurately estimate the Fisher Information of $\hat{r}$ with respect to the infectivity parameter κ, we follow the approach of Kallionatis et al. [47] which uses the Freedman-Diaconis rule [48] to determine the bin size $B=2\frac{R_{IQ}\left(X\right)}{n^{1/3}}$, where $R_{IQ}$ is the interquartile range, and *n* is the number of samples. Figure 11 demonstrates the Fisher Information of $\hat{r}$ with respect to the infectivity parameter κ.

We clearly observe a peak in the Fisher Information in close proximity to the critical value $\kappa^{*}$ relating to the onset of an epidemic phase in terms of global attack rates. This implies that the epidemic transition commonly associated with $R_{0}^{*}$ (and thus $\kappa^{*}$) has a specific spatial characteristic allowing it to be simultaneously understood as a percolation transition. We emphasize that this spatial percolation is observable in a realistic multi-agent heterogeneous setting, grounded in real data, complete with non-trivial and realistic transmission dynamics on a complex real-world topology of commuting and residential interactions distinct from the spatial network upon which the emergent clusters are identified.

## 5. Conclusions

In this paper, we studied influenza pandemics in terms of their spatial connectivity using the Australian Census-based Epidemic Model (AceMod), simulating influenza spread at a national level over an artificial population of ≈23.4 million agents, generated from the most recent 2016 Australian census data. It has been previously noted that Australian epidemics exhibit a relatively uncommon progression with characteristically bimodal epidemic curves [6]. Moreover, these modes have been associated with two distinct waves of infection upon the relatively separate urban and rural populations. Consequently, our analysis was tailored to this phenomenon, focussing on two key time points—the stationary points in mean local prevalence associated with peak urban and rural prevalence (see Figure 2 and Figure 3).

A primary finding, observable in both rural and urban peaks due to their high synchrony around the critical κ, is the behaviour of the infection connectivity, characterised in terms of percolation measures of clustering. By measuring this degree of clustering at various levels of infectivity, we have provided evidence, through direct computation of order parameters ($\langle \hat{r}\rangle$) and model free measures of criticality (the Fisher information), that the threshold transition is accompanied by a sharp increase in connectivity indicative of a percolation transition. As far as we are aware, this is the first time such a percolation transition has been implicated in a national, large-scale agent-based model of epidemics. We note that the location of this transition in terms of $R_{0}$ is lower than is generally observed in models based on homogeneous well mixed populations. This phenomenon is explained here and in other agent-based approaches by the extreme heterogeneity of individuals which interfere with the effectiveness of an average measure such as $R_{0}$, emphasising the importance of flexible, heterogeneous, modelling approaches such as agent-based modelling.

By examining both the statistical and individual behaviour of $\langle \hat{r}\rangle$ as both a function of time and of the infectivity, we have shown distinct clustering phenomenon associated with the urban and rural waves of the epidemic. In particular we observe the urban wave to be a consistent phenomenon with relatively constant behaviour beyond the critical point with relatively low variation in its timing and extent. However, the behaviour of the rural wave is much more nuanced. Of crucial importance is that, at all values of κ, the existence of a highly connected rural wave is not guaranteed, with it being a probabilistic phenomenon with relatively large variance. When the urban and rural peaks are distinguishable we see that the likelihood and extent of such a connected rural wave increases with increasing infectivity accompanied by an increase in the variance such that it is inherently less predictable than the urban wave. We conjecture that this is due to a fundamental sensitivity in the spread of the epidemic from urban to rural areas leading to a multiplicity of qualitatively distinct outcomes, though further research is needed on this issue.

Broadly, this study emphasises the importance of the bimodal, dual population behaviour of epidemics in Australia for both modelling and policy. Methodologically it also opens up interesting questions for further work, such as whether meaningful *local* measures of interconnectivity can be developed and applied to such complex systems allowing researchers to investigate the spatial patterns of infection on localized populations when there is low synchrony on the national scale. Furthermore, future work may focus on the development of warnings in real time based on the unique spatial properties and bimodal nature of the epidemic similarly to [1,2].

Finally, we mention the methodological developments that were necessary to perform this spatial analysis. An essential step required for the percolation analysis and subsequent assessments of criticality through the Fisher information is the dimensional reduction of the prevalence in local regions to strongly and weakly infected. This allows for canonical percolation measures to be utilized and ensures that computation of the Fisher information is tractable, but naturally comes at the expense of a certain degree of resolution. However, this loss in resolution has not affected the ability to observe the percolation phase transition and identify pertinent features of the secondary (rural) wave. We emphasise that the results presented here *utilize* the results of the AceMod simulation package, performing post-processing on the output formed of spatial time series. Consequently, all of the analyses may be equally applied to similar spatially labelled prevalence data from any national or disease context, whether it is simulation or empirical data. We thus hope that this contribution encourages the use of spatial analysis in the study and understanding of epidemic processes which may involve heterogeneity of demographics, mobility, and disease transmission factors more generally.
